# Subcellular Imaging of Liquid Silicone Coated-Intestinal Epithelial Cells

**DOI:** 10.1038/s41598-018-28912-x

**Published:** 2018-07-17

**Authors:** Peter Nirmalraj, Roman Lehner, Damien Thompson, Barbara Rothen-Rutishauser, Michael Mayer

**Affiliations:** 1 Adolphe Merkle Institute, University of Fribourg, Chemin des Verdiers 4, CH-1700 Fribourg, Switzerland; 2 Department of Physics, Bernal Institute, University of Limerick, Limerick, V94T9PX Ireland

## Abstract

Surface contamination and the formation of water bridge at the nanoscopic contact between an atomic force microscope tip and cell surface limits the maximum achievable spatial resolution on cells under ambient conditions. Structural information from fixed intestinal epithelial cell membrane is enhanced by fabricating a silicone liquid membrane that prevents ambient contaminants and accumulation of water at the interface between the cell membrane and the tip of an atomic force microscope. The clean and stable experimental platform permits the visualisation of the structure and orientation of microvilli present at the apical cell membrane under standard laboratory conditions together with registering topographical features within a microvillus. The method developed here can be implemented for preserving and imaging contaminant-free morphology of fixed cells which is central for both fundamental studies in cell biology and in the emerging field of digital pathology.

## Introduction

Surface contamination and formation of nanoscopic water bridges can reduce the spatial information content that can be gained through atomic force microscopy (AFM) measurements under ambient conditions. Specifically, water bridges tend to form at hydrophilic nanoscale contacts^1–3^ such as the interface between the tip of an AFM and a solid surface. To conduct high-resolution AFM imaging under ambient conditions (in air), it is crucial to limit the surface contamination and control the parameters governing formation of water bridges driven by capillary condensation at the tip-surface interface^4–6^. The thermodynamics of water bridge formation and rupture are well known^1,4,5,7^, including the influence of humidity^1^, radius of the probe^1,6^, distance between the probe and the sample^1^, pull-off force^5,6^ and frictional force^2,4^ on the water bridge structure. Supported by numerical models based on Kelvin equations^1,3^, density functional theory^7^ and Monte Carlo simulations^8^ these studies have argued that the water bridge fluctuates in response to changes in local physiochemical environment thereby limiting the maximum spatial resolution achievable from an AFM tool operating in aqueous media. Yet, aqueous solutions are required for sustaining and studying the sub-structure and dynamics of proteins and cells in their native state.

Previously, AFM studies have shown that it is possible to track protein motion^9^ and capture cell surface receptor interactions^10^ at room-temperature in aqueous media, which is an experimentally demanding environment when compared to cryogenic AFM measurements conducted in an ultraclean and thermally stable experimental platform. Although it is imperative to study biomolecular dynamics under physiological conditions, it has been shown that the intricate structures of fixed cells (in a dry state) can be better resolved with electron microscopy operating in vaccum^11^ than with AFM in water^12^. During electron microscopy studies of fixed cells the samples are protected from ambient contamination but at the cost of losing soluble cell contents^11^. Consequently, an alternative method is required, which leverages recent advances in instrumentation, preserves cellular morphology and simultaneously detects subcellular topological features without the requirement for multistep sample preparation procedures as in cryosectioning for AFM based cellular imaging^13^. Such a facile and robust methodology will have a direct impact on clinical level screening of pathological diseases^14^ characterised by cell structure malfunction, *e*.*g*., red blood cell diseases such as sickle-cell anaemia.

As proof of principle for high resolution imaging of cellular structures under ambient conditions, we demonstrate a method to resolve structural details of fixed microvilli on the apical domain of intestinal epithelial cells *in vitro*. Epithelial cells polarise when cultured to confluency on a petri dish or on a permeable membrane insert. The most characteristic structure of intestine epithelial cells consists of closely packed apical microvilli, which are also termed the brush border. The microvilli are important structures to increase the surface of enterocytes, are the primary site for nutrients absorption^15^ and are involved in mechanotransduction^16^. Here, we encapsulate the cell surface in a silicone liquid membrane^17^ fabricated by controlled spray deposition^17^ and employ an AFM probe (which is not fully immersed in liquid silicone), to image the silicone liquid-coated cellular surface. The silicone liquid enables stable AFM imaging under standard laboratory conditions by protecting the cell surface topology against ambient contaminants and minimising probe drift thereby providing a suitable platform for scanning probe microscopy measurements.

**Figure 1:**
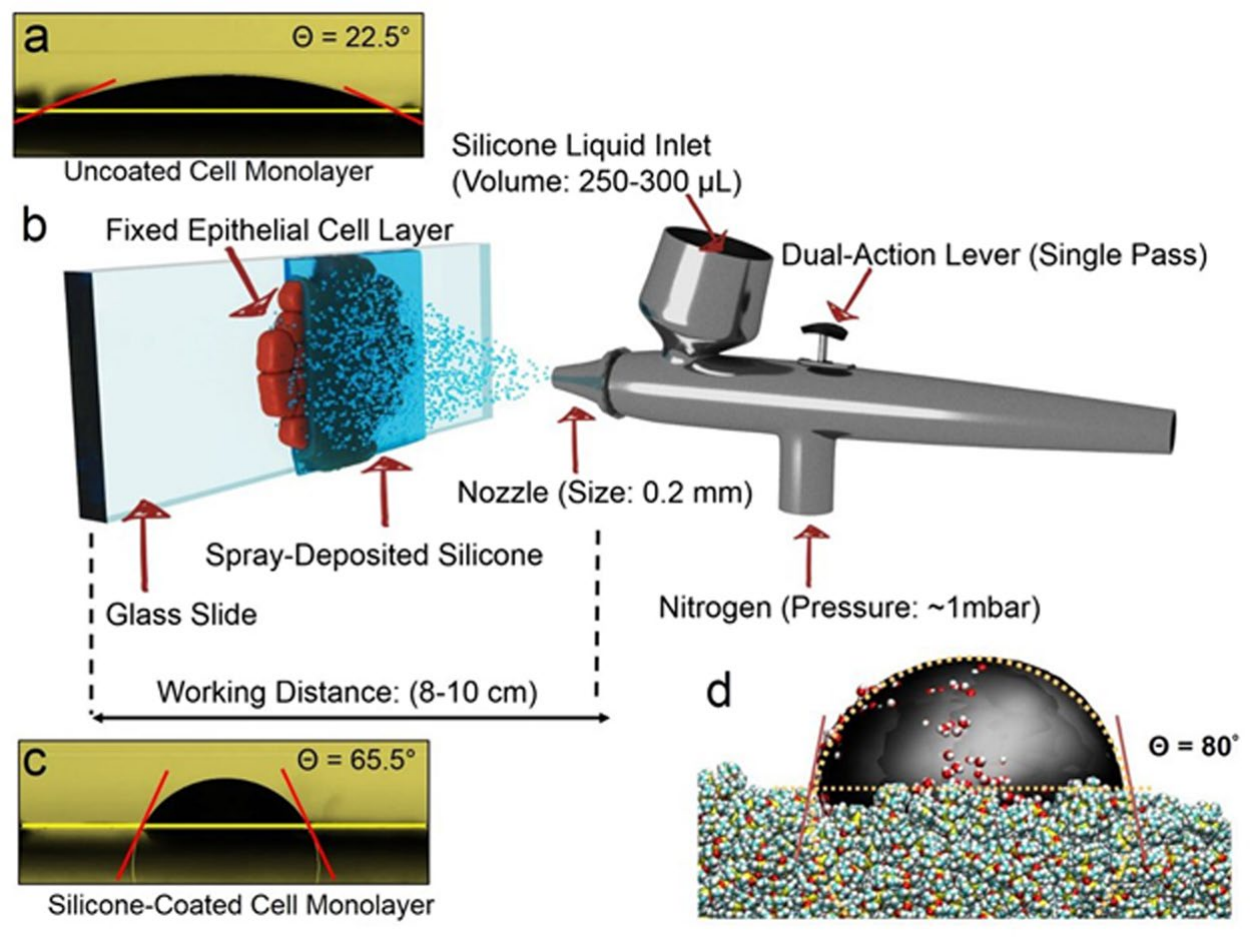
Fabricating liquid silicone membrane on fixed epithelial cell monolayer. (**a**) Water contact-angle measurements on a confluent layer of uncoated epithelial cells reveals a hydrophilic surface with a mean contact angle (Θ) of (23 ± 2.5)°. (**b**) Schematic of the spray-deposition process and parameters for fabricating the silicone membrane on top of the fixed epithelial cell layer (objects are not shown to scale). (**c**) Contact-angle measurements on the epithelial cell surface after spray-depositing the silicone membrane show an increased mean Θ of (63 ± 5)°, in qualitative agreement with the Θ of (80 ± 5)° calculated from (**d**) molecular dynamics simulations of the water droplet-silicone liquid interface.

A confluent Caco-2 intestinal epithelial cell monolayer with a well differentiated brush border (see Supplementary Figs S1 and S2) is a hydrophilic surface (Fig. 1a) with a water contact angle (Θ) of (23 ± 3)° averaged over ten locations on the cell layer. The morphology of the cells can degrade over time through interaction with ambient hydrocarbon contaminants^18,19^. To address this issue, we spray-coat the cell surface with liquid silicone to form a thin liquid membrane on top of the epithelial cell layer. Three key parameters optimised to create the silicone liquid membrane through spray-deposition as shown in the schematic (Fig. 1b) are the working distance between the spray nozzle and sample, pressure of compressed gas, and volume of the sprayed silicone liquid. A mean Θ of (63 ± 5)° was measured for the epithelial cells coated with liquid silicone (Fig. 1c) using the same protocol as for uncoated epithelial cell samples. The large increase in water contact angle from 23° to 63° shows that the silicone liquid coating significantly increases the hydrophobicity of the sample. Molecular dynamics (MD) simulations substantiate the large measured increase in contact angle, showing a calculated Θ of (80 ± 5)° on a silicone film (Fig. 1d) compared with a calculated Θ of (17 ± 3)° on an uncoated cell membrane (see Supplementary Fig. S3).

**Figure 2:**
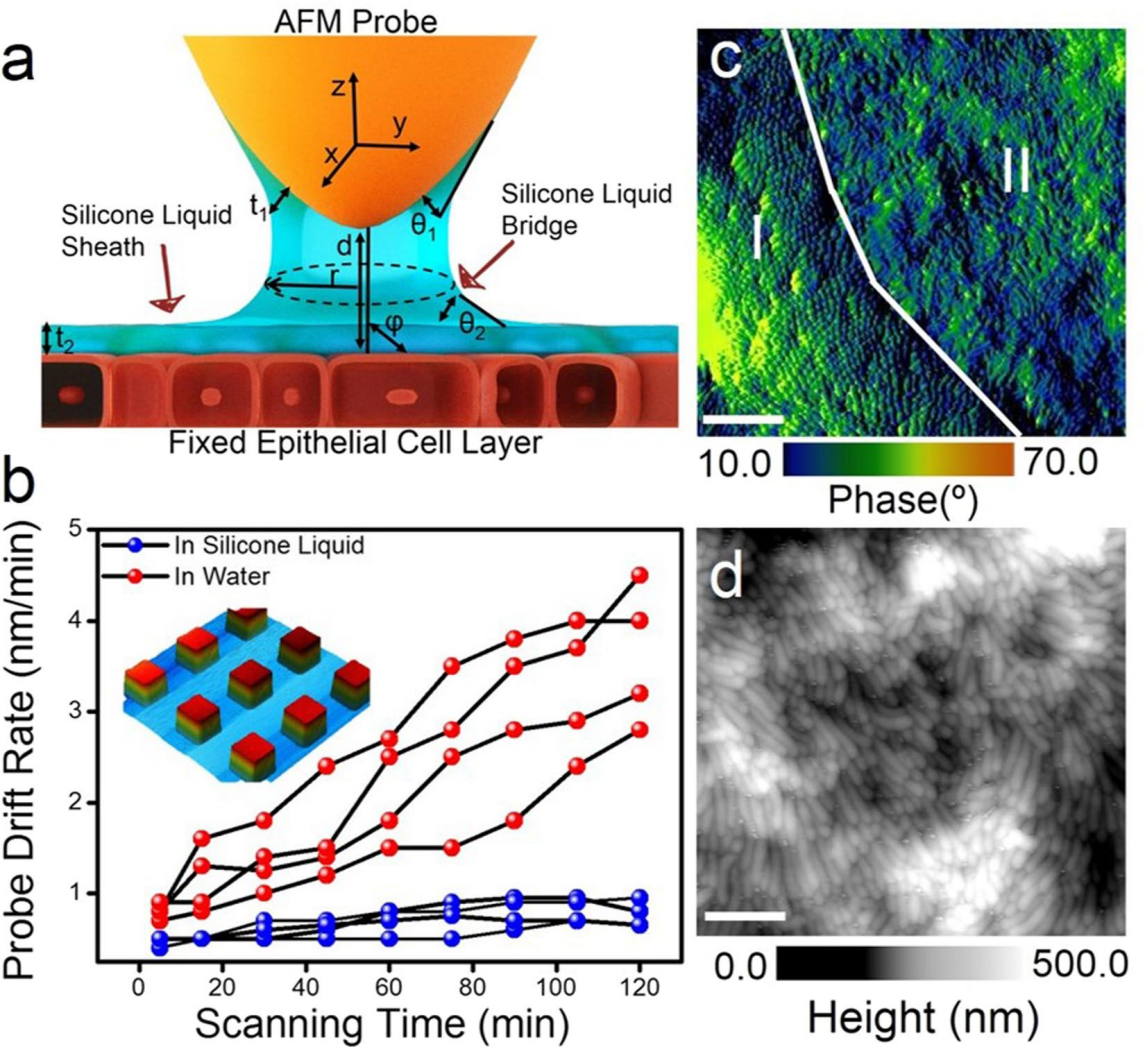
Imaging liquid silicone-coated cell monolayer. (**a**) Schematic of the silicone liquid bridge formed between the AFM probe and the silicone-coated epithelial cell surface. The silicone liquid membrane serves to protect both the hydrophilic cell surface and the probe apex against moisture and contaminant build-up. d is the distance of separation between the tip-apex and sample, r is the radius of the local meniscus and φ denotes the tilt angle of the meniscus. t_1_ and t_2_ and θ_1_ and θ_2_ are the thickness of the liquid membrane and the silicone liquid contact angle around the tip and on the sample surface, respectively. (**b**) Analysis of the rate of AFM probe drift as a function of experimental scanning time at (23 ± 1) °C when imaged over a Si calibration grid sample (shown in inset AFM image, scan size: 2.5 µm × 2.5 µm) in liquid silicone (blue curves) and water (red curves). The probe scan rate was maintained constant at 2 Hz for measurements in liquid silicone and water. (**c**) Large-area phase-contrast AFM image of liquid silicone-coated Caco-2 monolayer shows domains with distinct boundaries (indicated by white line). The perpendicular (marked by I) and parallel packing (marked by II) of microvilli with respect to the cell surface is visible from the phase-contrast AFM image (scale bar: 2.5 µm). (**d**) Spatially magnified AFM image of ordered microvilli structures oriented parallel to the cell surface (scale bar: 500 nm). AFM images shown in panel c (phase) and d (height) were acquired using intermittent contact mode in liquid silicone.

The ability to detect structural deviations in microvilli geometry is critical for determining their function during the absorption and release of nutrients^20^ and in coordinating immune responses^16^. To this end, we analysed microvilli structures on epithelial cells using an AFM probe in intermittent contact mode. First, we focus on the imaging stability of the AFM probe, where only the tip-apex is immersed in the silicone liquid (Fig. 2a). Figure 2b shows the drift rate of the AFM probe in silicone liquid (blue curve) and water (red curve) over two hours of scanning across a 2.5 µm^2^ area on a calibration grid sample (Fig. 2b inset) at a laboratory temperature of (23 ± 1) °C in intermittent contact mode. The near-constant probe drift rate of ≤1 nm per minute measured in silicone liquid at a scan rate of 2 Hz highlights the imaging stability when compared to the increasing probe drift rate measured in water using identical scan parameters. The low vapor pressure, high viscosity and low surface tension of silicone liquid circumvents the need to pump additional liquid through the flow-cell^21^, thereby improving the imaging stability, which is necessary for the extraction of intricate structural information from cells.

Figure 2c is a large-area AFM image recorded in phase-contrast mode of the epithelial cell surface during intermittent contact mode measurements. Physicochemical surface properties such as viscoelastic dissipation, adhesion and friction are captured by the phase image, which detects the lag in phase between the cantilever oscillation relative to the response of the cantilever^22^. The phase signal can register minute changes in surface topology that are difficult to resolve directly from the height signal^22^ (see Supplementary Fig. S4). The phase-contrast image (Fig. 2c) shows perpendicular (marked as I), parallel (marked as II, shown clearly in Fig. 2c) and random (see Supplementary Fig. S5) orientations of the microvilli relative to the epithelial cell membrane. These distinct packing modes are separated by a sharp domain boundary (traced as a white line in Fig. 2c). We calculate a mean root-mean-square (RMS) surface roughness value of (25 ± 5) nm for the perpendicular (region I) and (60 ± 8) nm for parallel (region II) structures, which reflect the differences in packing order and orientation within the microvilli domains. Repositioning the AFM probe (within region II) and reducing the scan area revealed an array of finger-shaped microvilli protrusions (AFM height image, Fig. 2d). The resolution of the data presented in Fig. 2d is comparable to previous AFM studies of microvilli structures on apical epithelial cell membrane in unfixed cells imaged in contact^23^ and peakforce tapping mode^12^ in aqueous media.

**Figure 3:**
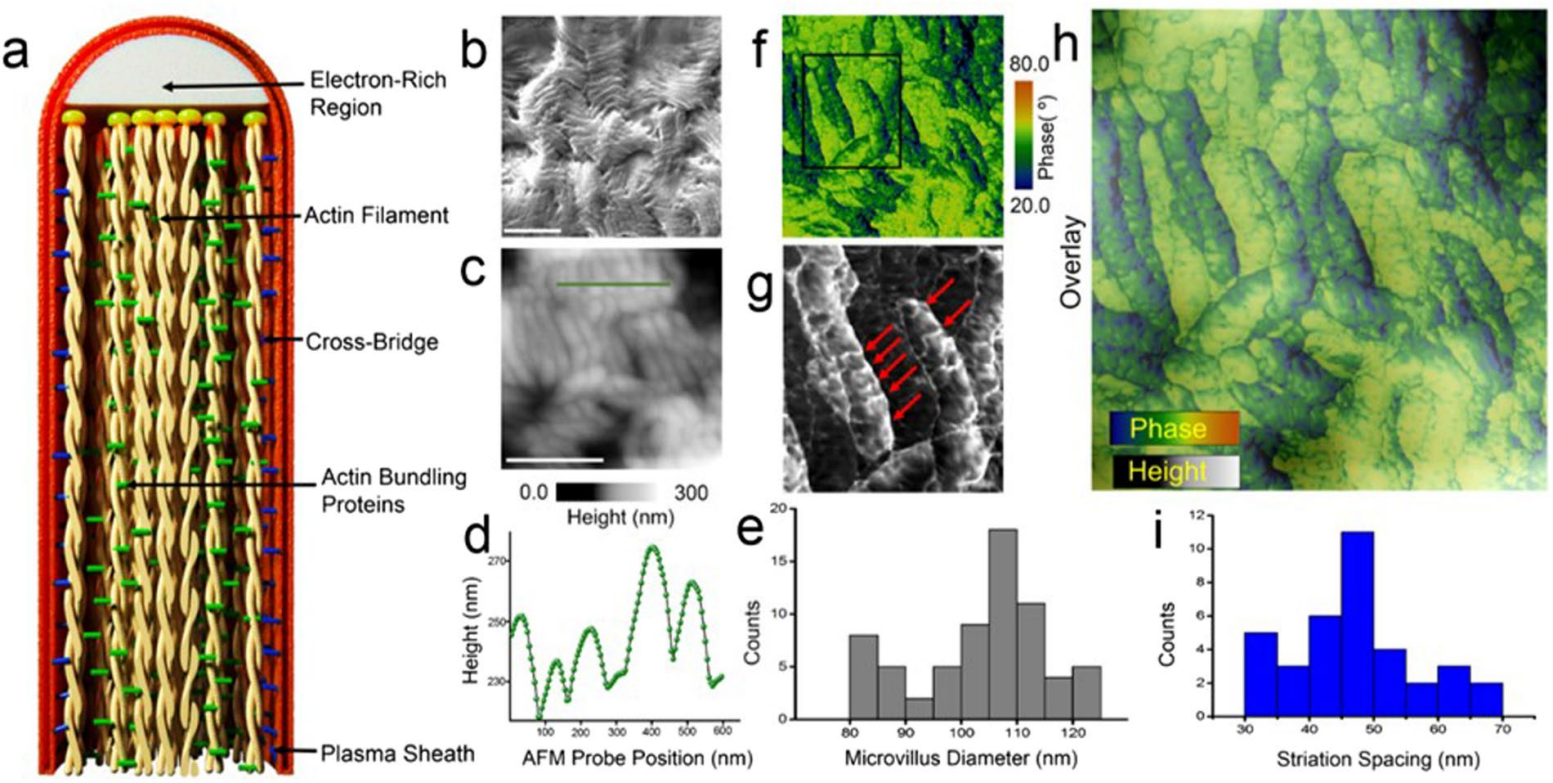
Resolving microvillus substructure. (**a**) Schematic detailing the internal structure of a microvillus tubule^25^ (features shown not to scale). (**b**) Scanning electron micrograph of the microvilli tubules (scale bar: 10 µm). (**c**) AFM topography of the ordered microvilli on epithelial cell membrane (scale bar: 500 nm). (**d**) Cross sectional analysis of the microvillus assembly along the line indicated in panel (a). The line profile reveals the nanoscopic height variations from which the diameter of a single microvillus tubule is determined as shown in the diameter distribution histogram (**e**). (**f**) The high-resolution phase contrast image corresponding to the height image shown in panel **c** shows intricate sub-structure details of the microvillus. (**g**) Spatially-magnified phase-contrast AFM image acquired over the region indicated by the black rectangle in panel (f) showing the striations marked by the red arrow. (**h**) Overlay of the height (panel c) and phase (panel f) data shows the nanoscale features on the surface of the microvilli (**i**) Statistical analysis of the striation spacings present along the length of the microvillus, the observed spacing is similar to previously reported spacing of cross bridges^11,24,25,27^ (see Supplementary Fig. S6 for line scan along the length of the microvilli showing the periodic spacing of the striations).

More detailed spatial information on single microvillus filaments (Fig. 3a) can be obtained by optimising the scanning parameters (see Methods section). For reference, Fig. 3b shows close-packed structure of the microvillus resolved by scanning electron microscopy (Fig. 3b). The AFM height image (Fig. 3c) and corresponding cross-sectional profiles (Fig. 3d shows a representative line profile) reveal the closely-packed finger-like microvilli structures with significant variations in local height and diameter. Averaging over multiple section analyses we calculate a mean microvillus diameter of 102 nm, with confidence interval lower (CIL) of 98 nm and confidence interval upper (CIU) of 104 nm (Fig. 3e). We calculate the confidence interval (CI) at 95% as the statistical distribution shown in Fig. 3e is non-gaussian. The mean microvillus diameter we report is consistent with pioneering high-resolution cryo-electron microscopy measurements^24–26^, where the diameter of a single actin filament is known to vary from 6–10 nm^11,25,27^, along the length of the microvillus^15,25^. The phase-contrast image reveals further details about the configuration of a single microvillus unit (Fig. 3f). In particular, we observe lateral striations along the entire length of the microvillus unit (previously observed only using high-resolution electron microscopy at cryogenic temperatures^24^), which are visible in the zoomed-in phase image (marked with red arrows in Fig. 3g) and in the overlay image (Fig. 3h). To the best of our knowledge, this level of detail on cellular structures in ambient conditions has not been previously obtainable using atomic force microscopy. The AFM phase data reveals a mean striation spacing of 46 nm with a CIL:42 nm and CIU: 50 nm based on the non-Gaussian statistical distribution shown in Fig. 3i (see Supplementary Fig. S6 for striation periodicity measured along a microvillus). Previous high-resolution electron microscopy studies on the structure of single microvillus have reported on similar lateral striations with a periodicity of 30–40 nm^11,24,25,27^, comparable to the striation periodicity measured here. These nanoscale structural features along the length of a microvillus have been attributed to the cross-bridge filaments whose periodicity can vary depending on the chemical environment in which the cells are fixed^24,25^.

The high spatial resolution of the microvilli fine structure obtained using atomic force microscopy in liquid silicone at room-temperature showcases a method to preserve fixed cellular features and visualise cell substructures. The method allows the direct imaging of nanoscale features both on cell surface and along the length of a single microvillus. This method is compatible with both commercially available standalone AFM tools and those used in conjunction with optical microscopy techniques^28^, which makes it amenable to field deployed screening of diseases^29^ related to cell malfunction. We anticipate that this microscopy based methodology will have an impact in the emerging field of digital pathology^30^, where it is important to register information on diseased cells in a fixed state that is free of ambient contaminants and of sufficiently high-spatial resolution for algorithm based data analysis.

## Methods

### Cell culture and preparation of fixed epithelial cells

Human colon (colorectal adenocarcinoma) cell line Caco-2 were obtained from American Type Culture Collection (ATCC, HTB-37) and cultivated at 37 °C under 5% CO_2_ water saturated atmosphere in complete medium consisting of Dulbecco’s modified Eagle medium (DMEM) (Gibco) supplemented with 10% non-heat inactivated fetal bovine serum (Gibco), 1% MEM nonessential amino acids (Gibco), 1% L-glutamine (Gibco) and 1% penicillin and streptomycin (Gibco). Routinely, Caco-2 cells were grown in 75 cm^2^ flasks (Corning, USA) and subcultured twice per week at a ratio of 1:3 or 1:4. Initially 4.5 × 10^5^ Caco-2 cells (passage 8–20) suspended in 0.5 mL of supplemented DMEM were seeded onto polycarbonate 12-well Transwell filters (Corning Incorporated, USA; 3 μm mean pore size, 0.9 cm^2^ surface area). Caco-2 cells were maintained under standard incubation conditions for 21 days and the medium on both the apical (0.5 mL) and basolateral sides (1.5 mL) changed every day. After cultivation in Transwell systems cells were washed twice with PBS. Fixation of the cells was performed in Karnovsky fixative (2% Paraformaldehyde, 2.5% Gluteraldehyde) for 2 h. Subsequently, cells were washed twice with PBS and dehydration was carried out through a graded series of ethanol (20–100%). Filter membranes were cut out and mounted on glass slides using carbon black tapes. Samples were dried by putting them into an exicator for 24 h.

### Fabrication of silicone liquid membrane

Silicone liquid (Sigma Aldrich, 317667-5 ML, CAS no: 63148-62-9), membranes were fabricated through a controlled spray deposition process using a Harder and Steenbeck dual action evolution airbrush in a fume cupboard with HEPA filters. The working distance between the spray nozzle and the sample surface was set at 8–10 cm, with a compressed gas (either air or nitrogen) pressure of ~1 mbar and a volume of silicone liquid of 250–300 µL. The silicone coated epithelial cells were structurally intact and free from ambient contaminants for 6–8 days (see Supplementary Fig. S5) whereas identically prepared samples when left uncoated showed signs of ambient contamination (see Supplementary Fig. S7). The water contact angle measurements were carried out using a Data analytics, QCA 15 pro tool.

### Intermittent contact mode AFM imaging in silicone liquid

AFM measurements were performed using a JPK Nanowizard II instrument with a flow-cell type liquid-cell chamber operated in intermittent contact (tapping) mode under standard laboratory conditions without any temperature or environmental control. We used a specialised AFM tip holder (JPK) that is compatible with liquid imaging. For the AFM tip we employed a commercially available probe from Budget Sensors, Multi 75-G, which is a monolithic uncoated silicon probe with an apex of <10 nm, force constant of 3 N/m and a nominal resonant frequency of 75 kHz when only the probe apex was immersed in liquid silicone, conditions under which the current study was conducted. To test the extent to which the resonant frequency of the tip can be reduced in silicone liquid medium, the cantilever and tip was fully immersed in liquid silicone and the resonant frequency of the tip was reduced to about 20 kHz, due to the damping of cantilever dynamics in dense liquid media^31^. Prior to imaging, the AFM cantilevers were cleaned by rinsing them in acetone for 30 sec followed by rising in isopropanol for 2 min to remove residual contamination from acetone and finally blow dried with compressed N_2_. The amplitude setpoint were adjusted to ~90% of the free amplitude of the cantilever and the high-resolution phase-contrast AFM images was registered when the ratio of drive amplitude and setpoint was adjusted to ~1. Before starting to engage the tip in silicone liquid environment the scan size and X,Y offset were set to 5 nm and 0, respectively, to avoid damaging the cell structure. Upon sensing the surface of the sample the scan size were increased in small steps. On engaging the amplitude setpoint, integral and proportional gains were routinely adjusted to maintain faithful tracking of cell surface structure with minimal error signal and noise. Initially, even after optimising the scan parameters in real-time, a constant background noise was detected from the trace-retrace curves, which persisted even after closing the hood to reduce acoustic noise and mounting the AFM tool on an active vibration cancellation table. Finally, we were able to identify the source of this background noise which stemmed from the dust accumulation in the light path of the AFM tool as pointed out by M.Stark *et al*.^22^, and by addressing this issue we were able to circumvent the background noise signal. For high resolution phase-contrast images, the drive amplitude is reduced as the free-amplitude goes lower than the setpoint and then the setpoint value is adjusted accordingly for better tracking of cell surface topology. This adjustment also helped in overcoming sticking effects of the tip on the sample surface. The phase-contrast images were obtained in both the net attractive (phase larger than 90^°^, softer tapping) and repulsive phase regime (phase lower than 90^°^, harder tapping) (see Supplementary Fig. S8). All AFM scans were collected at a line rate of 2–4 Hz at a resolution of 512 × 512 (that is 512 lines along the vertical direction and 512 sampling points on every line scan). For image processing, the raw data were analysed using Gwyddion 2.48 freeware (http://gwyddion.net) and subjected to first order flattening before calculation of surface roughness values from height images.

## Electronic supplementary material

Supplementary Information
