# Publisher Correction: Subcellular Imaging of Liquid Silicone Coated-Intestinal Epithelial Cells

**DOI:** 10.1038/s41598-018-32186-8

**Published:** 2018-09-27

**Authors:** Peter Nirmalraj, Roman Lehner, Damien Thompson, Barbara Rothen-Rutishauser, Michael Mayer

**Affiliations:** 1 Adolphe Merkle Institute, University of Fribourg, Chemin des Verdiers 4, CH-1700 Fribourg, Switzerland; 2 Department of Physics, Bernal Institute, University of Limerick, Limerick, V94T9PX Ireland

Correction to: *Scientific Reports* 10.1038/s41598-018-28912-x, published online 17 July 2018

The original version of this Article contained errors.

In the Abstract,

“The clean and stable experimental platform permits the visualisation of the structure and orientation of microvilli present at the apical cell membrane under standard laboratory conditions together with registering subcellular details within a microvillus.”

now reads:

“The clean and stable experimental platform permits the visualisation of the structure and orientation of microvilli present at the apical cell membrane under standard laboratory conditions together with registering topographical features within a microvillus.”

In the main text,

“In particular, we observe lateral striations running along the length of the microvillus unit (previously observed only using high-resolution electron microscopy at cryogenic temperatures^24^), which are visible in the zoomed-in phase image (marked with red arrows in Fig. 3g) and in the overlay image (Fig. 3h).”

now reads:

“In particular, we observe lateral striations along the entire length of the microvillus unit (previously observed only using high-resolution electron microscopy at cryogenic temperatures^24^), which are visible in the zoomed-in phase image (marked with red arrows in Fig. 3g) and in the overlay image (Fig. 3h).”

These errors have now been corrected in the PDF and HTML versions of the Article.

