# Supplementary material for: Subcellular Imaging of Liquid Silicone Coated-Intestinal Epithelial Cells
Source: Sci Rep. 2018 Jul 17;8:10763. doi: 10.1038/s41598-018-28912-x (PMC6050225; doi:10.1038/s41598-018-28912-x)
Supplement: Supplementary file 1 — Supplementary Information [file 41598_2018_28912_MOESM1_ESM.docx]

**Subcellular Imaging of Liquid Silicone Coated-Intestinal Epithelial Cells**

Peter Nirmalraj^1*^, Roman Lehner^1^, Damien Thompson^2^, Barbara Rothen-Rutishauser^1^ and Michael Mayer^1^.

1.Adolphe Merkle Institute, University of Fribourg, Chemin des Verdiers 4, CH-1700 Fribourg, Switzerland. 2. Department of Physics, Bernal Institute, University of Limerick, V94T9PX, Ireland.

*email: peter.nirmalraj@unifr.ch

*
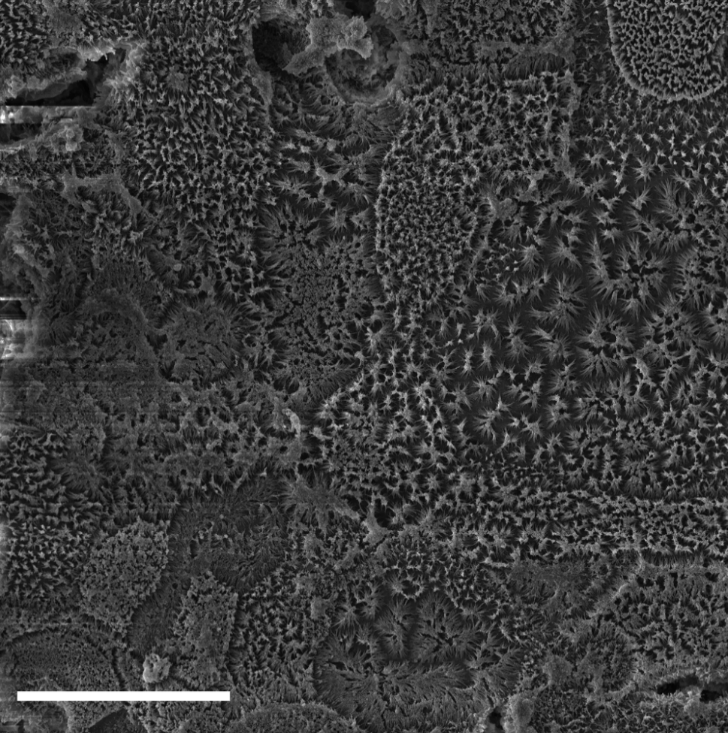
*

Figure S1 | Large area scanning electron microscope (SEM) image of fixed Caco2 epithelial cell layer using a TESCAN Mira3 LM field emission scanning electron microscope. Scale bar: 20 µm. The SEM data reveals densely packed microvilli regions forming a brush border. A spatially magnified SEM image of the microvilli structures is shown in Fig. 2b in the main manuscript.


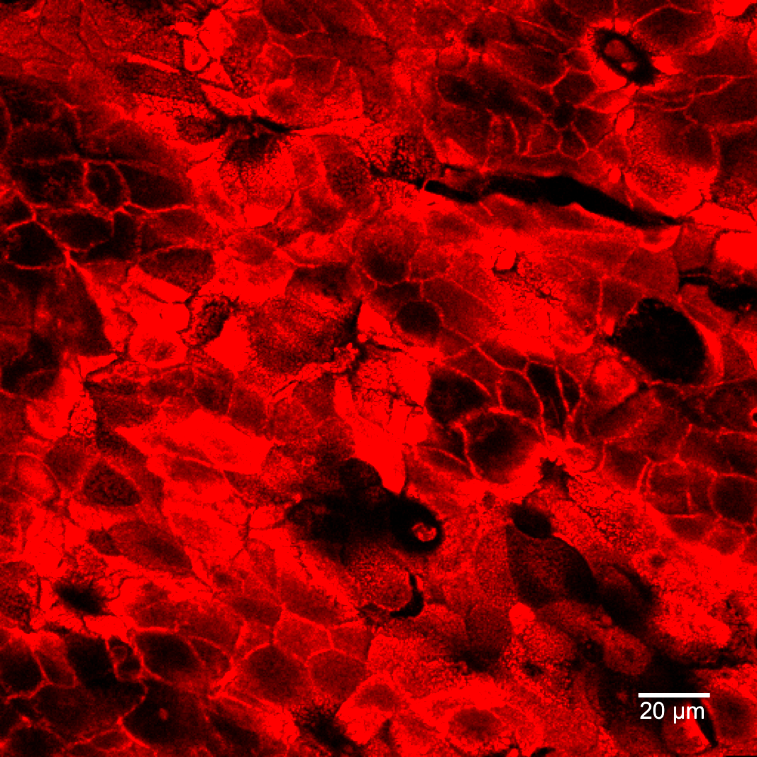


Figure S2 | Large area fluorescence light microscope (FLM) image of fixed Caco2 epithelial cell layer using a Zeiss LSM 710 microscope. The degree of labelling is relatively uniform, the red colored regions correspond to labelled actin filaments stained with rhodamine-b. The FLM image reveals well defined cells with cell-to-cell size variations.


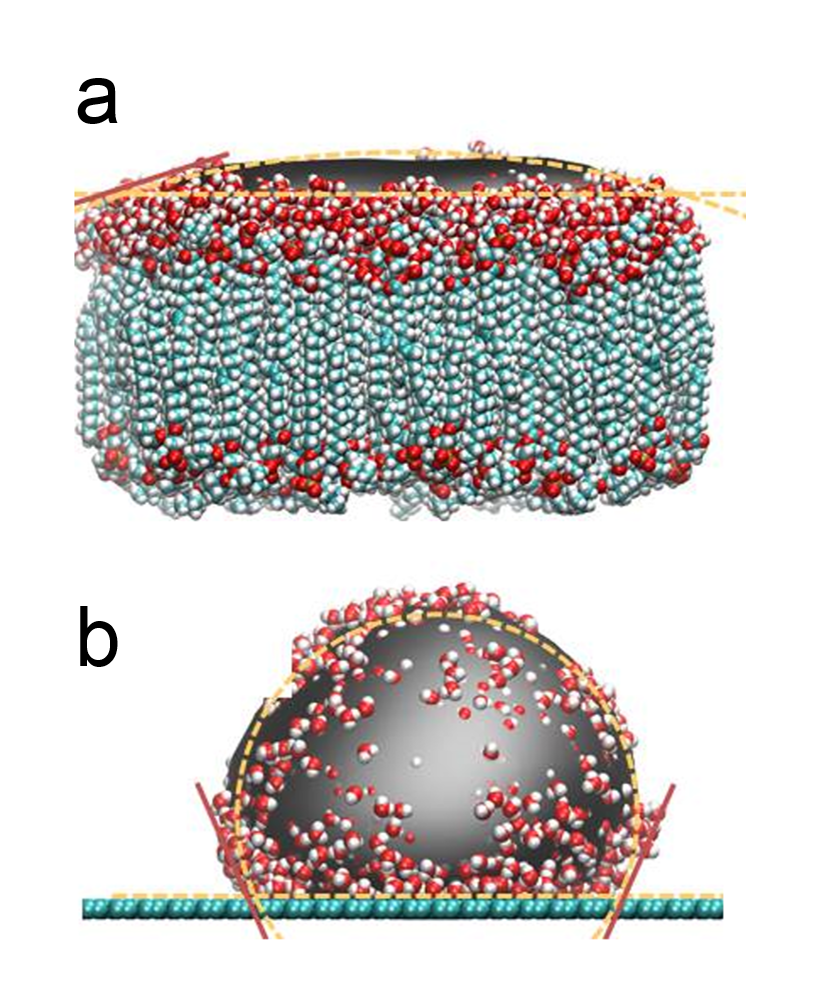


Figure S3 | Snapshot from molecular dynamics simulation of the formation of contact angle of water on lipid layers (a) and on hydrophobic graphene (b). Average values of 17±3 and 109±5 degrees, respectively, are calculated from 100 ns simulations using a water droplet containing ~3000 molecules and using the analysis methods and simulation parameters described previously^1-3^. These control simulations confirm that the layers of silicone liquid show wetting behaviour intermediate between the very hydrophilic cell membrane surface and very hydrophobic model atomic surface. The silicone oil model shown in main text Fig. 1d is composed of a ten-molecule deep layer of silicone oil polymers^3^ packed on a surface area of 10 x 10 nm^2^ and shows a calculated Θ of (80±5)° as the base of the water droplet is partially submerged in the thin silicone layer. Further control simulations (not shown) in which the silicone is allowed to deviate away from a layered structure showed a slightly smaller water contact angle of (72±6)° which is closer to the measured value of (63±5)°, indicating that the thin layer of silicone behaves as a more porous and so less hydrophobic material than bulk silicone oil (which has a measured Θ of 117°)^4^.


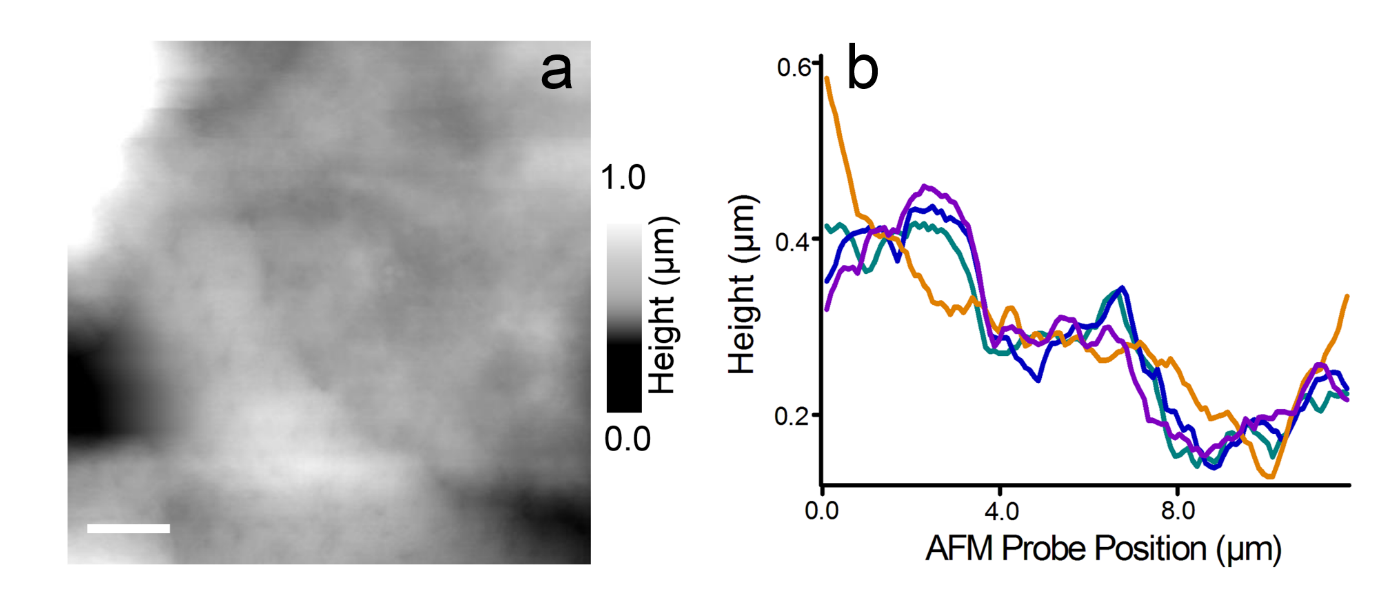


Figure S4 | (a) Large-area AFM image of the apical epithelial cell surface and the corresponding sectional analysis (b) acquired over four individual line scans on the epithelial cellular landscape. Scale bar: 2.5 µm.


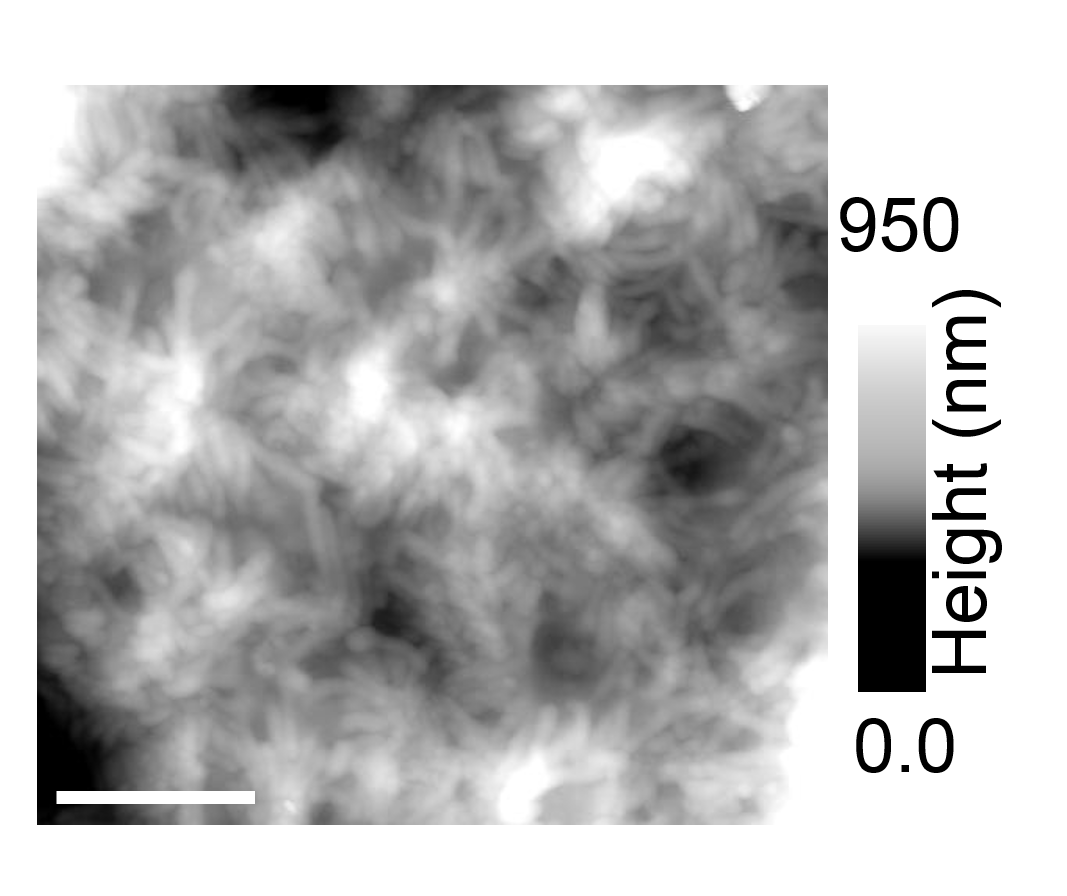


Figure S5 | AFM topographic image of randomly oriented microvilli structures imaged 6 days after mounting the epithelial cells to the glass slide followed by deposition of the silicone liquid membrane. Scale bar: 1 µm. The randomly oriented microvilli region has a mean surface roughness of (250 ± 80) nm which is significantly higher for perpendicularly (region I, Fig 1g main text) and parallelly oriented (region II, Fig 1g main text) microvilli structures. Even after 6 days of storage under ambient conditions, the sample surface shows no major indication of structural degradation as seen from the intact microvilli tubules or ambient contaminant, highlighting the role of the silicone liquid membrane in effectively shielding the cellular morphology.


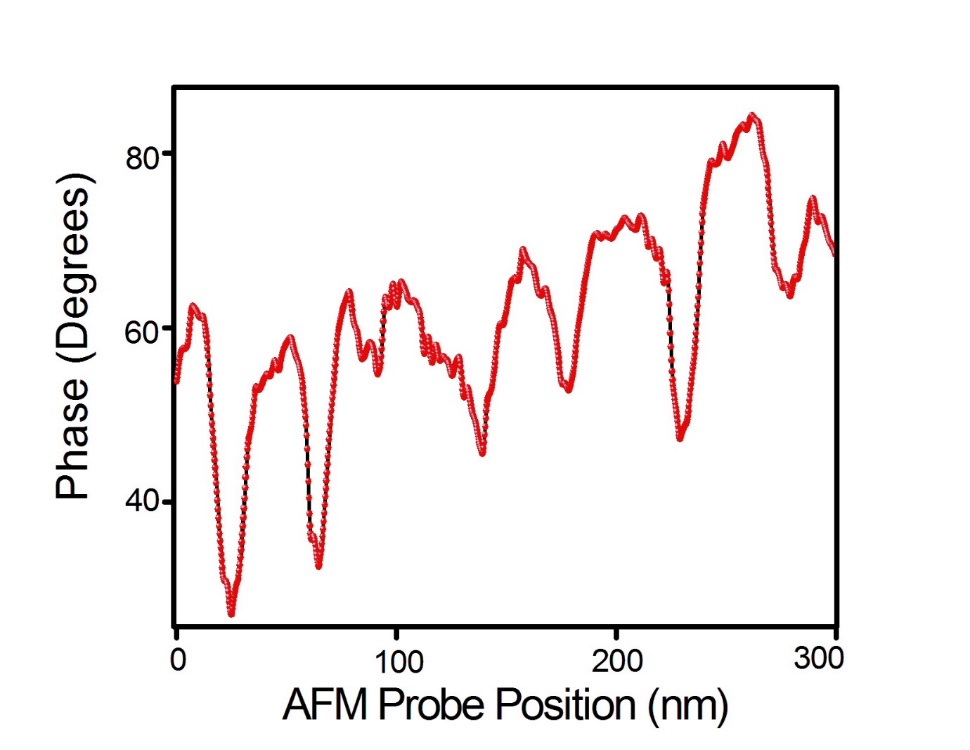


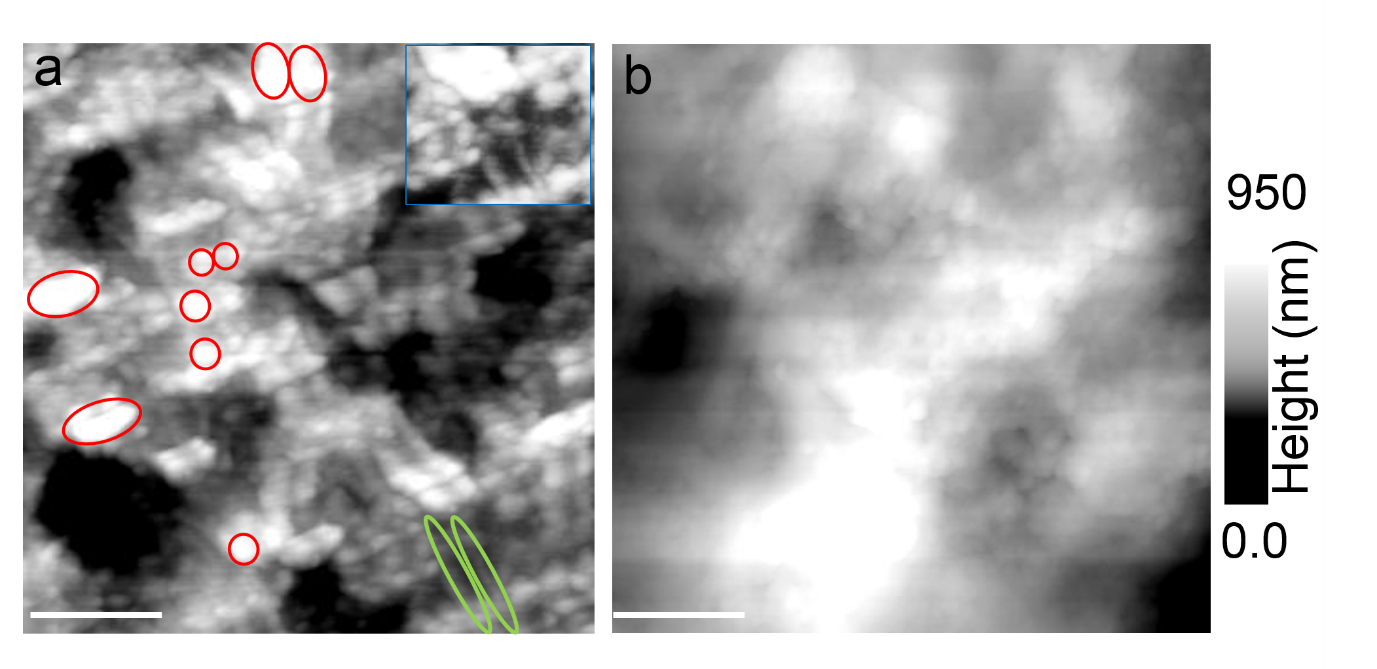
Figure S6 | Line scan along the length of the single microvilli structure registered in phase contrast AFM image (Fig. 2g main text) showing the periodically spaced striations.

Figure S7 | Topographic image of uncoated epithelial cell sample exposed to ambient air imaged under dry conditions. (a) AFM image recorded after 24 hours of direct sample exposure to ambient air. The red circles indicate ambient contaminants adsorbed on the epithelial cell layer where the microvillus tubules are visible (marked in green). Scale bar is 800 nm. The inset in panel **a** shows the microvilli structures marked by green. (b) AFM image recorded on the same sample after 6 days showing significant ambient surface contamination adsorbed on top of the cell monolayers. Scale bar is 800 nm. All the samples were stored under standard laboratory conditions.


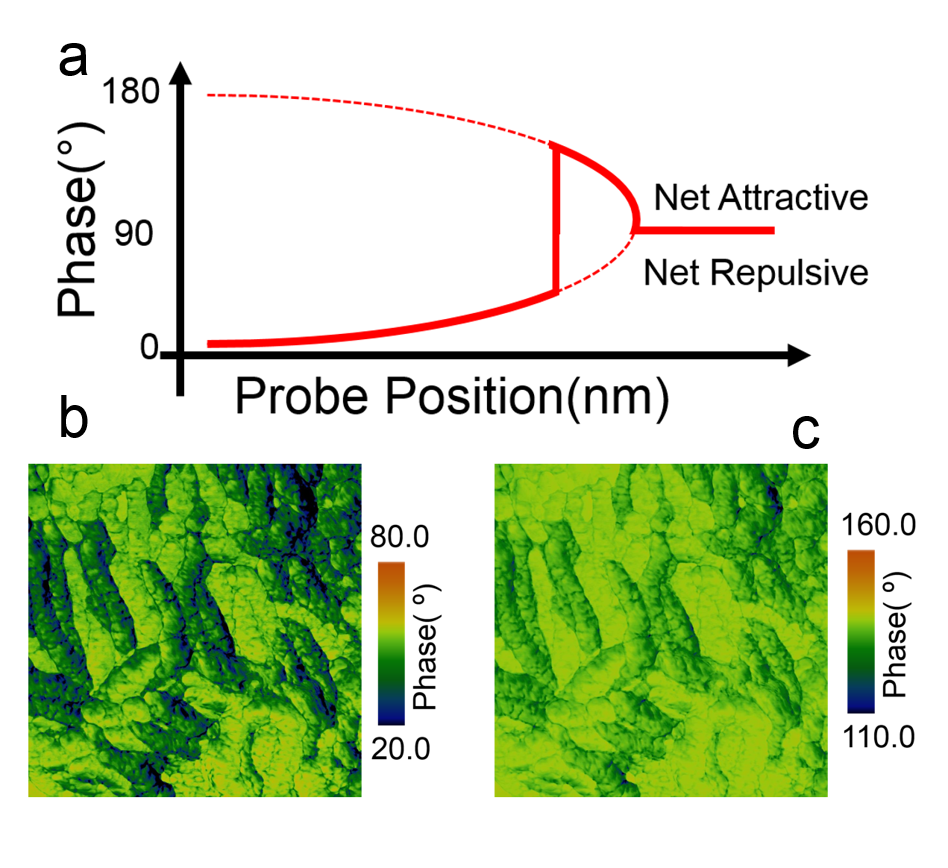


Figure S8 | Phase-contrast imaging in net repulsive and net attractive mode. (a) Schematic of net attractive and net repulsive force curve plot used in phase imaging. (b) and (c) Net repulsive (phase below 90^°^) and net attractive (phase above 90^°^) phase images acquired over the same microvilli region showing differences in local phase contrast.

**References**

1 Nawrocki, G. & Cieplak, M. Aqueous Amino Acids and Proteins Near the Surface of Gold in Hydrophilic and Hydrophobic Force Fields. *J Phys Chem C* **118**, 12929-12943, (2014).

2 Nawrocki, G., Cazade, P. A., Thompson, D. & Cieplak, M. Peptide Recognition Capabilities of Cellulose in Molecular Dynamics Simulations. *J Phys Chem C* **119**, 24404-24416, (2015).

3 Nirmalraj, P. *et al.* Nanoelectrical analysis of single molecules and atomic-scale materials at the solid/liquid interface. *Nat Mater* **13**, 947-953 (2014).

4 Chuah, Y. J. *et al.* Simple surface engineering of polydimethylsiloxane with polydopamine for stabilized mesenchymal stem cell adhesion and multipotency. *Sci Rep-Uk* **5**, 18162, (2015).
